# Automated Detection of Firearms and Knives in a CCTV Image

**DOI:** 10.3390/s16010047

**Published:** 2016-01-01

**Authors:** Michał Grega, Andrzej Matiolański, Piotr Guzik, Mikołaj Leszczuk

**Affiliations:** AGH University of Science and Technology, al. Mickiewicza 30, Krakow 30-059, Poland; matiolanski@kt.agh.edu.pl (A.M.); guzik@kt.agh.edu.pl (P.G.); leszczuk@kt.agh.edu.pl (M.L.)

**Keywords:** Haar cascade, OpenCV, pattern recognition, fuzzy classifier, data analysis, feature descriptor, knife detection, firearm detection

## Abstract

Closed circuit television systems (CCTV) are becoming more and more popular and are being deployed in many offices, housing estates and in most public spaces. Monitoring systems have been implemented in many European and American cities. This makes for an enormous load for the CCTV operators, as the number of camera views a single operator can monitor is limited by human factors. In this paper, we focus on the task of automated detection and recognition of dangerous situations for CCTV systems. We propose algorithms that are able to alert the human operator when a firearm or knife is visible in the image. We have focused on limiting the number of false alarms in order to allow for a real-life application of the system. The specificity and sensitivity of the knife detection are significantly better than others published recently. We have also managed to propose a version of a firearm detection algorithm that offers a near-zero rate of false alarms. We have shown that it is possible to create a system that is capable of an early warning in a dangerous situation, which may lead to faster and more effective response times and a reduction in the number of potential victims.

## 1. Introduction

Closed circuit television systems (CCTV) are becoming more and more popular and are being deployed in many offices, housing estates and in most public spaces. It is claimed that in the U.K., 1.85 to 4.2 million CCTV cameras are currently in operation (depending on the study) [1]. This makes for an enormous load for the CCTV operators, as the number of camera views a single operator can monitor is limited by human factors. According to the “CCTV Operational Requirements Manual 2009” [2], the task of the CCTV operator is to monitor and control, detect, observe, recognize and identify individuals and situations that are potentially harmful to other people and property.

A solution to the problem of overloading the human operator is to apply automated image-understanding algorithms, which, rather than substituting the human operator, alert them if a potentially dangerous situation is at hand.

When an individual carries a weapon (firearm or a knife) out in the open, it is a strong indicator of a potentially dangerous situation. While some countries allow for open carry firearms, in such an event, it is still advisable to grab the CCTV operators’ attention in order to assess the situation at hand.

During recent years, an increase in the number of incidents with the use of dangerous tools in public spaces can be observed. Starting with the USA and then in Europe, so-called active shooter incidents took place. Such an incident is a case when an armed individual or a small group of individuals attacks a random group of victims with the goal of wounding and killing as many as possible. The most notable incidents were those at Columbine High School (USA, 37 victims), the attack on Uotya Island by Andreas Breivik (Norway, 179 victims) or the attack by the Islamic fundamentalists at the Charlie Hebdo newspaper (France, 23 victims). According to the report published by the FBI [3] in 2013, in the time period between 2000 and 2013, there were 160 such incidents resulting in 1043 victims in the USA alone.

Automated methods for video surveillance have started to emerge in recent years, mainly for the purpose of intelligent transportation systems (ITS). They include traffic surveillance [4] and recognition of cars [5]. In this study, we have focused on the specific task of automated detection and recognition of dangerous situations applicable in general for any CCTV system. The problem we are tackling is the automated detection of dangerous weapons—knives and firearms, the most frequently used and deadly weapons. The appearance of such objects held in a hand is an example of a sign of danger to which the human operator must be alerted.

Our research was further motivated by our experience with the end users. While designing the algorithms, we received interest and remarks from European Police forces, local authorities and companies that deploy city-wide monitoring systems. It is worth mentioning that a vote carried out in 2014 among the residents of Krakow, Poland (approximately one million inhabitants), has obliged the local authorities to deploy a city-wide CCTV monitoring system. The city council advisory board has explicitly advised the implementation of a so-called “smart” monitoring system, capable of automated detection of threatening situations.

Krakow is not the only Polish city implementing monitoring systems. The Wroclaw (also approximately one million inhabitants) video monitoring system has been operating since 2009 (the monitoring system consists of 103 cameras). The cameras managed to stop many of the perpetrators of acts of vandalism. The system developed in Poznan (approximately 500 thousand inhabitants), since 2000, currently has more than 450 cameras. The monitored areas showed a decrease in crime, according to police data: fights and beatings by 40%, drug offenses by approximately 60%, as well as a general decline in vandalism and pick-pocketing. Bydgoszcz (approximately 400 thousand inhabitants) has implemented a system consisting of 84 PTZ and 50 stationary cameras.

Among other European cities, it is worth mentioning London (approximately eight million inhabitants), where there is estimated to be over approximately 900,000 cameras, of which the police have access to 60,000 (Heathrow Airport alone is monitored by some 3000 cameras). Monitoring in London is considered a valuable source of information about crimes. In 2009, Scotland Yard used CCTV recordings during the investigation in 95% of the cases of murder. In contrast, Glasgow’s (approximately 600 thousand inhabitants) monitoring system, which is operated by “Glasgow Community Safety”, jointly owned by the City Council and the Scottish Police, has only 500 cameras.

In the U.S., Chicago (approximately three million inhabitants) has implemented a monitoring system with 22,000 cameras. Despite the initial reluctance of residents to install surveillance cameras when their use started bringing tangible benefits, the attitude of society has greatly improved. The use of monitoring resulted in statistically-significant improvement in security in the areas monitored, measured as the incidence of crime. Houston (approximately two million inhabitants) has almost 1000 cameras, which are accessible to the police; the number of cameras is growing steadily. Dallas (approximately one million inhabitants) has 40 cameras in the city center (31 of them are PTZ cameras; nine others are stationary). Cameras have been installed in other areas selected by the police (the police have 140 such cameras) as areas where crimes are committed most often. It was found that only 6% of the area of the city is responsible for 40% of the crimes committed. After installing the cameras, since 2007, there was a 35% to 60% decrease in the number of crimes committed in monitored areas. Baltimore (approximately 600 thousand inhabitants) has about 700 cameras, and the system has been developed since 2005. It has been observed that crime fell by 15% in the regions monitored, and the system allows the police to make more than 1200 arrests per year. Similar systems were also launched in Philadelphia and San Jose.

Active shooter events in Europe and the U.S. have shown that their detection and recognition lead to a rapid response and a reduction in the number of casualties. Automated alarms can never hope to substitute the human operators of such systems; however, they may be useful, especially in a situation where a single operator monitors numerous CCTV cameras for many hours, which naturally dulls their awareness and ability to assess the situation.

In [6,7,8,9,10,11], we propose an initial approach to systems designed for knife and firearm detection in images, respectively. In this work, we summarize this effort and present the current versions of the algorithm. Even if different methods are also used, the algorithms presented in this paper aim towards a similar goal; our motivation is to solve the problem of knife or firearm recognition in frames from camera video sequences. The aim of these approaches is to provide the capability of detecting dangerous situations in real life environments, e.g., if a person equipped with a knife or firearm starts to threaten other people. The algorithms are designed to alert the human operator when an individual carrying a dangerous object is visible in an image.

We present the complex problem of fully-automated CCTV image analysis and situation recognition. We define the requirements for a fully-automated detection and recognition solution, and we propose a complex, multi-stage algorithm and evaluate its effectiveness and limitations in given conditions. Finally, we discuss the results and point to further development paths for our solution and similar techniques.

The remainder of this paper is structured as follows. Section 2 provides information on related work. Section 3 presents the methods.

## 2. Related Work

The concept of automated image understanding from video for public security applications is well known and well explored in many domains. For example, Jang and Turk proposed a system for vehicle recognition based on the SURF feature detection algorithm [12].

The concept of automated CCTV image analysis and detection of dangerous situations has been proposed and analyzed in several studies. Marbach *et al.* proposed a system for automated fire detection based on the temporal variation of fire intensity [13]. This and similar solutions exploit a similar research direction, while dealing with a less complex problem.

This is also the case for systems designed for observation and deduction based on human silhouette detection and pose estimation. A good overview of silhouette representation is proposed by Chen *et al.* in [14]. Such an approach is used in the crowd density management system proposed by Velastin *et al.* [15] and the congestion detection system proposed by Lo *et al.* [16]. Dever *et al.* proposed a system for automated robbery recognition based on actors’ pose estimation [17].

The initial concept of automated detection of gun crime was proposed by Darker *et al.* as part of the United Kingdom-based MEDUSAproject [18]. This team also worked on identifying the cues that might indicate that an individual is carrying a concealed firearm [19]. The first experiments made by the same team for utilizing CCTV as an automated sensor for firearm detection emerged next [20]. An example of a more recent approach is FISVER, a framework for smart public safety in video-surveyed vehicles, which has the ability of general object detection, including objects, such as firearms [21]. Furthermore, Arslan *et al.* proposed a solution for threat assessment using visual hierarchy and conceptual firearms ontology [22]. A good overview of the current progress in automated CCTV surveillance systems is presented by Dee and Velastin in [23].

Furthermore, it should be noted that there are other promising approaches in the detection of dangerous objects in similar scenarios. Yong *et al.* have shown that it is possible to detect metal objects, such as guns and knives, using microwave swept-frequency radar [24]. Objects can also be recognized using X-ray imaging, as shown by Mery *et al.* [25]. The practical application of such approaches is limited by the economic cost and health hazards. In addition, video-based firearm detection is a preventive measure with respect to acoustic gunshot detection and can be coupled with it [26,27].

Our approach was based on several tools designed for object detection and recognition. We have successfully applied MPEG-7 visual descriptors both in this work and in other research directed towards safety-related applications and computer forensics. Examples include the INACT Tool (an intelligent, advanced image cataloging tool [28] for combating child abuse) and the INSTREET Tool (an application for urban photograph localization [29]). Detection of dangerous objects is a specific case of general object detection, which can be carried out using methods, such as principal components analysis (PCA) [30], which is also applied in this work.

## 3. Methods

The starting point for designing algorithms for knife and firearm detection was a requirement analysis. We analyzed publicly-available CCTV recordings featuring crimes committed using a dangerous object. Several observations were made:

- real-life CCTV recordings are usually of poor quality, suffering from blurriness, under- and over-exposure, compression artifacts and others
- real-life CCTV recordings are usually of low resolution due to the poor quality of inexpensive CCTV cameras
- the dangerous object is visible only for a limited period of time in a scene, remaining hidden by the perpetrator most of the time.

Based on these observations, we have created a set of requirements for our systems. First, we decided that our algorithm needs to cope well with poor quality input. This means a low resolution input image and a small size of the dangerous object (in pixels). We also decided that the algorithm should work in real time utilizing no more than a typical desktop computer and without the need for specialized hardware, such as access to a supercomputing center or parallel computing.

One of the most important points is to keep the number of false alarms as low as possible (high specificity), even at the cost of missing some events (at the cost of sensitivity). This is due to the fact that if an automated algorithm generates too many alarms, the operator starts to ignore them, which, in turn, renders the whole system useless. Moreover, an algorithm that misses some events is obviously better than running the system blind (without any smart capabilities). False alarms are unacceptable in practical application due to the high costs they generate, as each alarm has to be verified by a human operator, causing stress and overload. Still, while maintaining a low number of false alarms, we try to achieve as high a sensitivity as possible.

Finally, following discussions with CCTV system retailers and operators, we have designed the system to be a sensing and supporting system, rather than a decision making one. This means that each time a dangerous object is detected, the human operator has to be alerted in order to assess the situation and take appropriate action. This is due to the fact that such an automated system is not capable of assessing the context and, thus, the potential severity of the situation at hand.

### 3.1. Knife Detection

**Figure 1:**
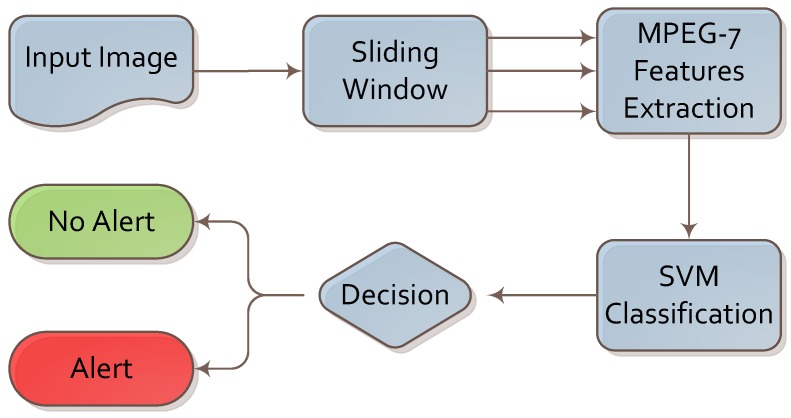
Algorithm for knife detection.

We designed the knife detection algorithm based on visual descriptors and machine learning. The complete flow of the proposed algorithm is presented in Figure 1.

The first step was to choose image candidates as cropped sections from the input. We chose candidates using a modified sliding window technique. In contrast to the original sliding window, we looked for knives near the human silhouette only and when at least one human silhouette appears in the image. We believe that a knife is only dangerous when held by a person. In addition, detecting a knife held in the hand in a limited part of the image is faster. Furthermore, a hand holding a knife has more characteristic visual features than a knife on its own, so we can expect better results. We distinguished two areas in the image: one near the potential offender and the other close to the potential victim. In those areas, we can expect the knife to show due to the general dynamics of a knife attack. Usually, a knife is held in the hand and used against the body of another person.

**Figure 2:**
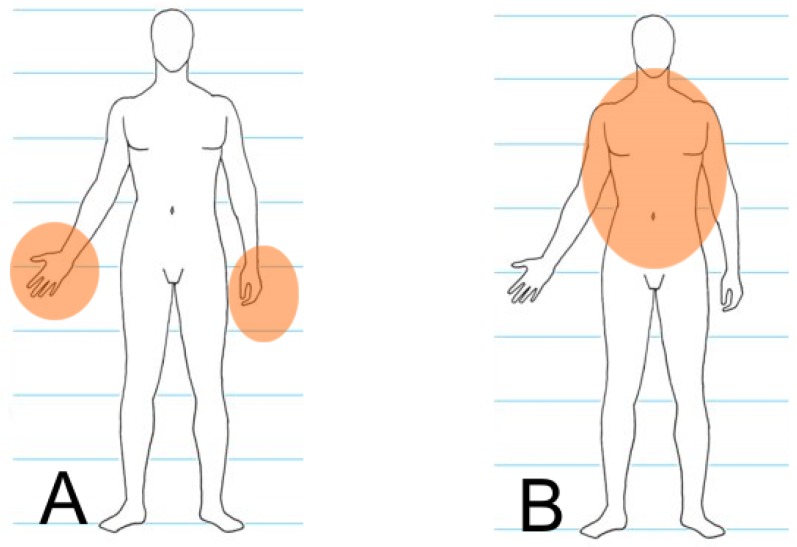
Areas where a knife may appear near offender (**A**) and defender (**B**) silhouettes.

It is impossible to distinguish between the offender and the defender automatically during processing because of the dynamics of such events. For this reason, both areas are observed for each human silhouette found in the image (each human silhouette is considered to be both a potential offender and a potential victim). The model is presented in Figure 2, and the whole process is discussed in detail in [31]. Any further considerations use the above assumptions for preparing the dataset described in Section 4.

The next step was to convert the image into its numerical representation. We are using a sliding window mechanism to find parts of images that contain features that are characteristic for knives. This way, we are able to determine the approximate position of the knife in an image. We do not need to detect the knife’s edges, which is not trivial when images with a variable and non-homogenous background are considered. The current literature describes many different visual descriptors along with their advantages and disadvantages [5]. We chose to use visual descriptors from the MPEG-7 standard. Due to the specific knife image pattern, we chose two descriptors: edge histogram [32] and homogeneous texture [33]. The first contains information about various types of edges in the image. It is a numerical vector that contains counts of eighty different types of edges. The second describes specific image patterns, such as directionality, coarseness and regularity of patterns, in the image. The two descriptors provide complex information about features characteristic of knives (edge, peak and steel surface of the blade). The edge histogram and homogeneous texture descriptors are represented by vectors of 80 and 62 elements, respectively.

The edge histogram defines five edge types. There are four directional edges and a non-directional edge. The four directional edges include vertical, horizontal, 45-degree and 135-degree diagonal edges. These directional edges are extracted from the image blocks. If the image block contains an arbitrary edge without any directionality, then it is classified as a non-directional edge. To extract both directional and non-directional edge features, we need to define a small square image block. Applying edge detection filters described in [32], the edge strengths for five edge types are calculated. The extraction procedure is widely described in [34].

The homogenous texture characterizes the region texture using mean energy and energy deviation from a set of frequency channels. The mean energy and its deviation are computed in each of 30 frequency channels [35]. The energy $e_{i}$ of the *i*-th feature channel is defined by Gabor-filtered Fourier transform coefficients derived using Formulas (1) and (2). The energy deviation $d_{i}$ of the *i*-th feature channel is defined in a similar form by Formulas (3) and (4). The extraction procedure is described in detail in [34].

(1) $$e_{i}=log_{10}\left[1+p_{i}\right]$$

(2) $$p_{i}=\sum_{\omega =0^{+}}^{1}\sum_{\theta ={\left(0{}^{\circ}\right)}^{+}}^{360{}^{\circ}}{\left[G_{s,r}\left(\omega ,\theta \right)∥\omega ∥P\left(\omega ,\theta \right)\right]}^{2}$$

(3) $$d_{i}=log_{10}\left[1+q_{i}\right]$$

(4) $$q_{i}=\sqrt[{}]{\sum_{\omega =0^{+}}^{1}\sum_{\theta ={\left(0{}^{\circ}\right)}^{+}}^{360{}^{\circ}}{\left\{{\left[G_{s,r}\left(\omega ,\theta \right)∥\omega ∥P\left(\omega ,\theta \right)\right]}^{2}-p_{i}\right\}}^{2}}$$

We avoided using color and keypoint-based descriptors because of the many potential distortions and errors. Color-based descriptors are unable to deal with light reflections and different color balances of image sensors. Keypoint-based descriptors were also unsuitable for the problem, since knives do not have many characteristic features. More keypoints were frequently detected around the object rather than on the knife itself. Because of the great number of different types of knives, we decided on similarity-based descriptors rather than those based on keypoint matching or exact shape. The numerical representations of the descriptors were stored as binary vectors for shorter access time and easier processing. The feature vectors are used in the decision making part of the system.

The extracted feature vector is an input to a support vector machine (SVM). We used *ν*-SVM with the decision function given by:

(5) $$f\left(x\right)=sgn\left(\sum_{i}\alpha_{i}y_{i}k\left(x,x_{i}\right)+b\right)$$

We used a nonlinear version of this algorithm with Gaussian radial basis functions (RBF) given by:

(6) $$k\left(x,x^{\prime }\right)=e^{-\lambda {||x-x^{\prime }\left|\right|}^{2}}$$

as a kernel. This algorithm alongside the appropriate optimization problem and its solution is described in detail in [36]. To find the best SVM parameters, we used a simple grid search algorithm guided by four-fold cross-validation results. The final decision about the alert is made based on the SVM result.

### 3.2. Firearm Detection

In order to assess different approaches to the problem of firearm detection, we conducted a series of proof-of-concept experiments. Following the initial experiments, we observed that it is extremely difficult to create an algorithm for this task that fully meets all of the requirements. We decided that several simplifications were necessary in order to meet the requirements.

- We decided to focus on a single type of firearm: a pistol.
- The dataset was gathered in a controlled environment, as almost all algorithms used for image recognition are sensitive to changing light conditions, such as day/night transitions. The footage was filmed indoors, which eliminated from the algorithm the potentially confusing motion of tree branches, birds, grass, and so on.

**Figure 3:**
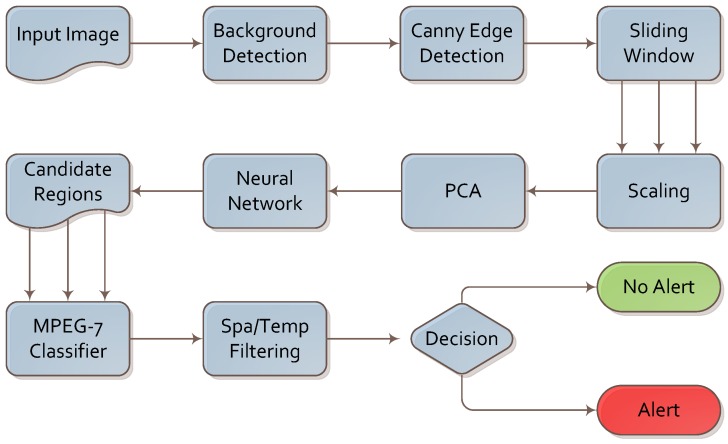
Algorithm for firearm detection.

The algorithm for firearm detection is presented in Figure 3. We analyzed the footage frame by frame; however, the final decision was based on both intra- and inter-frame analysis.

**Figure 4:**
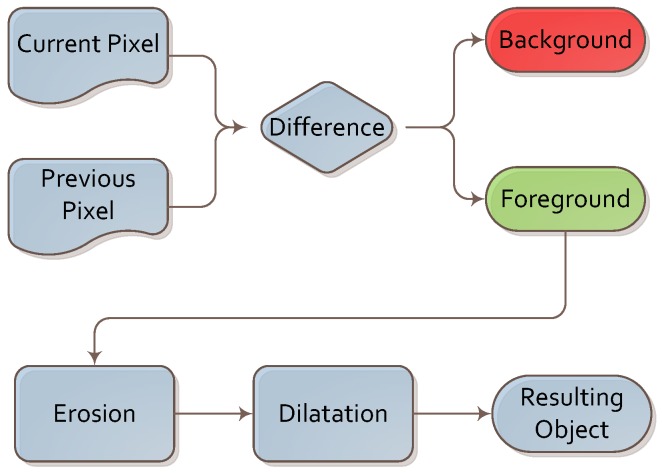
Algorithm for background subtraction.

A simple background subtraction algorithm was executed first. It was based on image differences between consecutive frames. As image differences leave multiple artifacts due to image flickering and changes in illumination, we supported it with two simple operations: erosion and dilation. These two operations allowed us to remove these artifacts and focus further steps of the algorithm on the foreground part of the image. This sub-algorithm is depicted in Figure 4.

This algorithm was chosen for its simplicity, low computational power requirements and good performance. We conducted the experiment indoors, so as to not have to deal with interference from small moving elements, such as tree branches or patches of light and shade. In such conditions, we found that a simple frame difference algorithm (with averaging across past frames) was sufficient. We plan to experiment with different background extraction algorithms (such as those based on predictive filtering or a Gaussian mixture model [37]) in future work.

Next, the Canny edge detection [38] algorithm was implemented in order to convert the image into a set of edges. This algorithm was applied only to the foreground region detected in the previous step in order to conserve computational power. This algorithm was chosen after comparing its performance and results with Harris, Sobel and Laplace filtering.

In the next step, samples of the image were taken using the sliding window technique. The image was analyzed multiple times with the increasing size of the sliding window. The size of the sliding window needs to be altered, as the distance of the object (firearm) from the camera influences the size of the object in the frame. We selected the optimal sliding step value, sliding window size and size increment following a series of experiments. This technique is very costly in terms of performance, even when applied to the foreground region only.

During the research, we analyzed an interesting option for limiting the number of input images from the sliding window. We used a depth camera capable of obtaining a depth image of the scene. We applied this to locate and identify the body and limbs of a person in the frame. Having identified the limbs, we focused the analysis on the area around the person’s hands. While this approach gave a performance boost in terms of computing requirements and accuracy, we did not pursue it for practical reasons. For the depth camera, we used the Microsoft Kinect. Unfortunately, this device has serious limitations: it has a range limited to a few meters, and it uses an infrared projector for depth measurement that cannot be used outdoors. For this reason, we assessed such a solution as impractical and decided not to use depth information and limb detection in this study.

In the next step, the samples obtained by the sliding window were scaled to a common size of 40 × 30 pixels creating a vector of 1200 values. We removed any samples containing a low number of edges (lower than 11%) as non-informative.

The scaled samples are fed into the PCA [39] method in order to reduce the dimensionality of the input vector to 560 values. We discovered that this step allows us to trade off 3% of the sensitivity and specificity for a four-fold increase in computational speed per frame.

The 560-value vector is fed into a three-layer neural network (NN). The NN was constructed using 560 neurons in the input layer, 200 neurons in the hidden layer and nine neurons in the output layer. Eight of the nine outputs are activated in the case of detection, depending on the spatial orientation of the dangerous object. The 9th output neuron is activated if no dangerous object is detected. The NN was trained using 1000 positive and 3500 negative examples from the training set (described in Section 4). The early stopping method was used to cope with the low number of training examples (when compared to the size of the NN). We observed that such a network provides us with high sensitivity and low specificity; therefore, the samples chosen by the NN are treated as candidate regions for further analysis.

In the next step, we use the MPEG-7 region shape (RS) descriptor [34,40,41] to compare the shape found in the candidate region selected by the NN with a generic firearm descriptor created from the positive examples in the training set. The RS descriptor has 140 bits, which contain 35 angular radial transform (ART) coefficients. An ART $F_{nm}$ coefficient is derived using Formula (7) where f(r,θ) denotes the intensity function in polar coordinates and $V_{nm}^{*}\left(r,\theta \right)$ denotes the ART basis function with the order of nm (in this case n=10 and m=10).

(7) $$F_{nm}=\int_{2\pi }^{0}\int_{1}^{0}V_{nm}^{*}\left(r,\theta \right),f\left(r,\theta \right)drd\theta$$

The ART function consists of parts that are separable (Equation (8)) in angular (Equation (9)) and radial (Equation (10)) directions.

(8) $$V_{mn}\left(r,\theta \right)=R_{n}\left(r\right)A_{m}\left(\theta \right)$$

(9) $$R_{n}\left(r\right)=\left\{\begin{array}{cc} 1, & \left(n=0\right)\\ 2\cos(\pi nr) & \left(n>0\right) \end{array}\right.$$

(10) $$A_{m}\left(\rho \right)=\frac{1}{2\pi }e^{jm\theta }$$

We use the Euclidean metric to compare the descriptor calculated for the sample with the generic one. If the distance is smaller than an experimentally-chosen threshold, we treat the sample as a true positive. The threshold was chosen in a series of experiments on the training test set.

Finally, spatial and temporal filtering was applied. We assumed that a firearm is normally visible in a series of consecutive frames and that it will not move significantly across the image. If a knife is detected in a set number of consecutive frames and within a certain range from the initial detection, an alarm is raised. We chose the values for the spatial and temporal filter experimentally using the training set.

**Figure 5:**
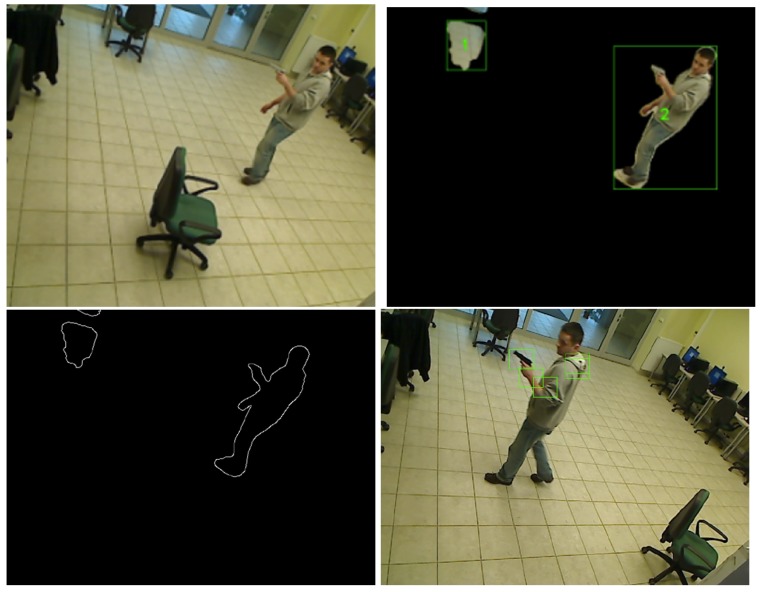
Image processed with background detection and Canny edge detection algorithms. (**a**) Input image; (**b**) Background detection; (**c**) Canny edge detection; (**d**) Neural network output.

The output images from selected steps of the algorithm are presented in Figure 5.

## 4. Training and Test Sets

Adequate datasets are mandatory elements of research. While there are no publicly-available datasets for CCTV research on firearms with a suitable number of examples, we made the decision to create our own datasets. Taking different methods into account, we created independent datasets for each problem (knife and firearm detection).

### 4.1. Dataset for Knife Detection

The dataset for knife detection was obtained from CCTV recordings. The images were cropped from the original frames using the sliding window method. The window size W × H was intentionally set to 100 × 100 pixels each. Such small image examples suit the condition and quality of real CCTV recordings, which are often of poor quality and blurred, and the object is small. In addition, our previous research shows that the size of the image in a dataset has a marginal effect on the final classification results [10].

The database consists of two classes of images (positive and negative examples).

- Positive examples (PE): A knife held in a hand is visible in the image. Only a knife held in a hand is considered to be a dangerous situation. We consider a knife not being held by a person to be less dangerous. It can also be easily omitted during processing or result in many false alarms.
- Negative examples (NE): A knife does not appear in the image. NE outnumber PE to cover as many cases as possible. NE images were taken under similar conditions as PE images.

**Figure 6:**
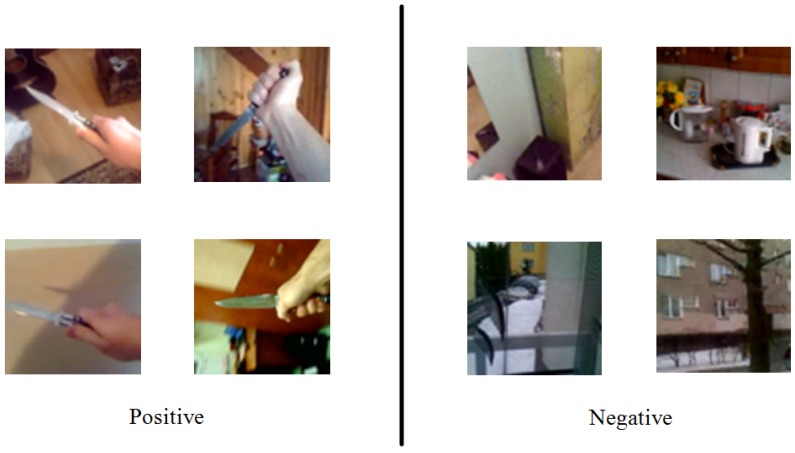
Sample images from the knife detection dataset: positive and negative.

The whole dataset consists of 12,899 images divided into 9340 NE and 3559 PE images. Some images were taken indoors, while some were taken through a car window in the street (because holding a knife openly in a public place is forbidden by law in Poland). A few sample images are presented in Figure 6. The complete image database is available for download at [42].

The dataset is not divided arbitrarily into learning and test sets. To avoid mistakes caused by badly-prepared sets, we used the cross-validation algorithm during the processing of our experiment.

### 4.2. Dataset for Gun Detection

In order to detect guns in CCTV recordings, training and testing test sets were prepared. Both test sets were created by shooting a series of CCTV recordings with an actor. This was because we were unable to obtain a sufficient number of real-life video shots among publicly-available clips. The complete video database is available for download at [43].

For the training set, positive examples for training the algorithms were manually selected from the frames containing the firearm. Additionally, negative training samples were selected from frames in which no firearm was visible. For the testing set, each frame containing a firearm was marked, including the region of the frame where the firearm was present. These tasks enabled us to create a detailed training set and a well-marked testing set for assessing the sensitivity and specificity of the algorithms.

**Figure 7:**
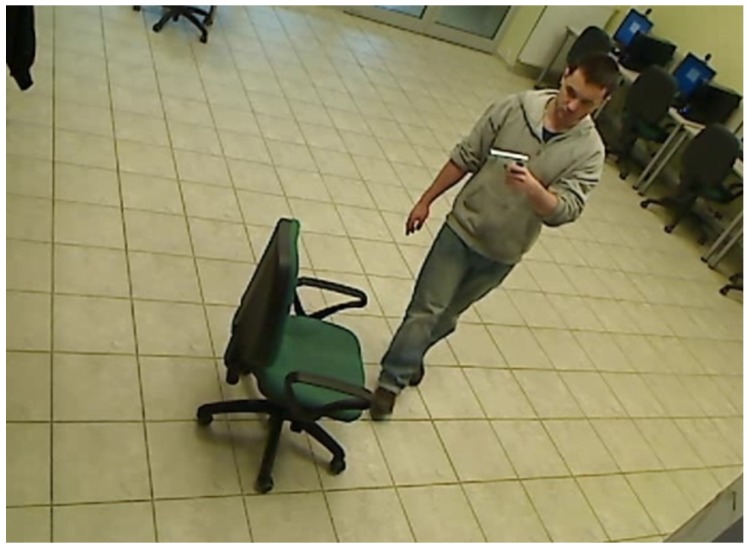
A frame from a dataset movie. Note the poor quality, small size and low contrast of the firearm against the background.

The training and testing set were the same size, with 8.5 min of recording resulting in approximately 12,000 frames each. Sixty percent of each set consisted of negative examples (not containing a firearm, but containing other objects being held in a hand), while 40% contained positive examples (a firearm visible to an observer). The size of the set was limited by the tediousness of the manual marking of the firearm in each frame. A frame from one of the movies is presented in Figure 7.

## 5. Results

This section presents results for both of our algorithms. In each case, we use similar measures to describe the results of the evaluation.

### 5.1. Results for Knife Detection

The knife detection algorithm was trained and tested on the dataset described in Section 4. To find the best SVM parameters, we used a simple grid search algorithm guided by four-fold cross-validation results. We found that the best results were obtained for γ=0.006 and ν=0.1 for edge histogram features and γ=0.00005 and ν=0.4 for homogeneous texture features, where *γ* is a kernel parameter and *ν* is a parameter controlling the number of support vectors. For feature extraction, we used the MPEG-7 library [44], which provides methods for generating edge histogram and homogeneous texture descriptors.

The results are presented in four tables: Table 1 and Table 3 for the edge histogram descriptor and Table 2 and Table 4 for the homogeneous texture descriptor. The edge histogram is shown to be better at solving the knife detection problem. The large number of true negatives and just 5% false positives means that the number of false alarms is reduced to a minimum. The accuracy reaches 91% when edge histogram features are used. Sensitivity and specificity reached 81% and 95%, respectively. The results are significantly better than others published recently. Algorithms tested on the same dataset [7,10,45] achieved 86%, 77% and 79% accuracy, respectively. Compared to other methods, the proposed algorithm also results in better accuracy than, e.g., [46]. The homogeneous texture descriptor returns significantly worse results. However, in our opinion, it can still be used to filter out false alarms thanks to its low false alarm rate (7%) and relatively high specificity (93%). The figures provided for false alarm rates were obtained with SVM returning only the most probable class (in this case, either knife or non-knife). In real CCTV systems SVM may be easily parametrized to lower the false alarm rate, *i.e.*, by shifting the probability threshold for the detection of a knife towards higher values. In such a case, the solution is going to have significantly higher specificity (arbitrarily close to 100%) at the expense of lower sensitivity. In real CCTV systems, low sensitivity means that the knife will not be detected in every single frame. This is not an issue because of a relatively huge number of frames containing a knife, even if it appears only for a few seconds.

**Table 1:** Knife detection: results for the edge histogram descriptor.

	Positive	Negative
True	81.18%	94.93%
False	5.07%	18.82%

**Table 2:** Knife detection: results for the homogeneous texture descriptor.

	Positive	Negative
True	52.95%	93.00%
False	7.00%	47.05%

**Table 3:** Knife detection: results for the edge histogram descriptor.

Number of Examples in Test Set	2627
Sensitivity	81.18%
Sensitivity	94.93%

**Table 4:** Knife detection: results for the homogeneous texture descriptor.

Number of Examples in Test Set	2627
Sensitivity	52.95%
Sensitivity	93.00%

The solution to the knife detection problem deals with poor quality and low resolution images; this is important given the fact that several video artifacts may appear in live streaming applications [47], affecting people detection [48]. Many CCTV systems only provide a certain quality of footage. It should be noted that the algorithm is processed in real time.

### 5.2. Results for Firearm Detection

The results of the algorithm were assessed on the test movies accompanied by an accurate per-frame description. This allowed us to conduct a precise estimation of the algorithm metrics. We conducted a test for two test recordings. In the first recording, the actor was holding a firearm; however, the firearm was not visible for the whole duration of the recording. For periods of time, it was occluded by the actors’ body or it was out of the camera’s coverage.

In the second test recording, the actor did not carry a firearm. Instead, he was bare handed or was holding a casual item, such as a bag or a folded umbrella.

The results for the test movies containing and not containing a firearm are presented in Table 5.

**Table 5:** Firearm detection: results for the base version of the algorithm.

	Movie with Firearms	Movie without Firearms
Number of frames	4425	7920
Sensitivity	95%, 18%	n/a
Sensitivity	95%, 58%	99%, 32%

While the results presented in Table 5 seem to be excellent, they do not meet the requirements we set for the system. For the movie that does not contain a dangerous object, we obtain a specificity of 99.32%. While numerically, this is an excellent result, in practice, it means that we obtain approximately 50 false positives in the whole sequence. This renders such a system unusable in a real scenario, as the operator would be overwhelmed with false alarms. From our research and discussions with potential end-users of such systems, we know that trading sensitivity for specificity is fully acceptable, meaning that the algorithm will miss out on some of the dangerous events, but will not generate false alarms.

For this reason, we tweaked our system by changing the temporal filtering parameters, so that the number of false positives for the sequence without a dangerous object would reach zero at the cost of reduced sensitivity. We then applied the tweaked algorithm to the movie containing the dangerous object. The results are presented in Table 6.

**Table 6:** Firearm detection: results for the algorithm with a reduced number of false alarms.

	Movie with Firearms	Movie without Firearms
Number of frames	4425	7920
Sensitivity	35%, 98%	n/a
Sensitivity	96%, 69%	100%

While we noted a significant drop (from 95% to 35%) in sensitivity for the movie containing dangerous objects, at the same time, we achieved a specificity of 100% for the movie not containing the objects. Although it misses a significant number of frames with dangerous objects, the algorithm generates no false alarms for a movie without a firearm, thus becoming a useful and valuable CCTV aid. In our solution, there are still numerous false alarms for movies containing dangerous objects. However, considering the difficult visual conditions, the result is satisfactory.

## 6. Conclusions

In this study, we focused on the two specific tasks of automated detection and recognition of dangerous situations. We have proposed, implemented and tested algorithms for the detection of a dangerous tool held in a hand. A knife or a firearm (the most frequently-used weapons in assaults) held in a person’s hand is an example of a sign of danger.

The specificity and sensitivity of the knife detection algorithm are 94.93% and 81.18%, respectively. These results are significantly better than others published recently. Our solution to the knife detection problem deals with poor quality and low resolution images. This is important because many CCTV systems only provide such quality of footage. It should be noted that the algorithm is processed in real time.

For the firearm detection algorithm, we achieved a specificity of 96.69% and a sensitivity of 35.98% for the video containing dangerous objects, and we noted a specificity of 100% for the movie not containing dangerous objects. Although it misses a significant number of frames with dangerous objects, the algorithm generates no false alarms, thus becoming a useful and valuable CCTV aid. In our solution, there are still numerous false alarms for movies containing dangerous objects. However, considering the difficult visual conditions, the result is satisfactory.

We plan to continue our work on the algorithms in order to provide a complete and ready-to-market solution for CTTV operators. We intend to conduct more tests in three defined scenarios. The “bank” scenario is an indoor situation in which the camera is close to the perpetrator and the scene is well lit. The “street” scenario, on the contrary, has to cope with poor light conditions and the high distance of the person from the camera, resulting in low resolution of the objects to be recognized. We also plan to integrate both algorithms into a single solution, while further focusing on reducing false alarms and increasing sensitivity. Another research direction that we will pursue is the introduction of new modalities: the introduction of pan-tilt-zoom cameras, the infrared spectrum for low light conditions and thermography for better distinction of the dangerous tool from the background. We also foresee extending the number of detected classes by other firearm types and by other dangerous objects (e.g., machetes, clubs and bats).
